# Case of a Large Tumor Involving the Superior Maxillary Bone, Removed by Professor Pancoast at the Clinic of the Jefferson Medical College

**Published:** 1848-08

**Authors:** James V. Patterson


*Case of a Large Tumour involving the Superior Maxillary Bone, removed by* Prof. Pancoast *at the Clinic of the Jefferson Medical College.* Reported by James V. Patterson, M. D.

Rachel Flora, æt. 18, a strong, healthy coloured girl, residing in Bucks Co., Pa., had suffered from the presence of an epulis tumour of the upper jaw for the last ten years.

When she was two or three years old, and then living in Virginia, she had a tumour of the same description removed from her upper jaw by a physician of that State; but after an interval of three or four years it returned again, and has been gradually increasing, until it has attained its present size. The tumour as it now appears, extends from the anterior part of the superior maxillary bones, across the mouth, as far back as the epiglottis, being attached in front to the alveoli of the incisor teeth, and behind to the palatine processes of the superior maxillary, filling up the cavity of the mouth, and materially interfering with respiration and deglutition, so that for the last few months the patient has been nourished entirely by liquids. In its character, the tumour evidently belonged to that class of tumours known under the name of erectile, vascular in its nature, swelling up and pulsating forcibly under the least excitement of the circulation, so as to threaten the patient with suffocation. Only a few days after her arrival in this city, she had an attack of hysteria, during the paroxysm of which, the tumour swelled up to such an extent, as to endanger her life by suffocation, and to demand the free use of the lancet.

As the removal of such a vascular mass would necessarily give rise to a great amount of hemorrhage, it was deemed expedient and requisite to tie the primitive carotid artery of that side; for the ligation of the external carotid alone would not suffice, as there existed such a free anastomosis between the vessels of the external and internal carotids, secondary hemorrhage would be likely to take place, which might prove fatal. Accordingly, on the 6th of November, Prof. Pancoast, in the presence of the class, performed the operation for ligation of the primitive carotid on the left side, after the usual manner, and succeeded in throwing a ligature around the artery, though the struggles of the patient and the great fear lest she should have a hysterical convulsion during the operation, made this a rather difficult undertaking. Everything went on favourably, and in the course of two weeks she was prepared to have the whole tumour removed.

On the 20th of November, the patient having been brought into the amphitheatre, Prof. Pancoast removed the whole of the diseased mass after the following manner:

The face being placed opposite a strong light, and the head of the patient supported against the breast of an assistant, an incision was made through the cheek, from the left angle of the mouth, in a semicircular direction, towards the outer canthus of the eye, after the manner of Velpeau's operation, so as to avoid wounding the parotid duct and the portio dura nerve.

The flap was then rapidly dissected back from the bone, and with a small saw the superior maxillary was divided transversely by an incision parallel to the alveolar processes, then with a strong pair of cutting forceps a vertical incision was made through the bone at each end of the transverse, one by the side of the last incisor tooth on the right side, and the other by the last molar tooth of the left, and the mass, thus isolated from its bony attachments, was quickly removed. The hemorrhage following was restrained by the use of the actual cautery; and the wound having been plugged with lint, the lips of the incision were united by hare-lip suture and adhesive strips, and the patient placed in bed, and ordered to live on a liquid diet.

The wound in the cheek healed up by first intention in five days, and, on the tenth day after the operation, the condition of her mouth was such that she was enabled to eat meat, a thing which she had not done before for several months. The wound of the jaw rapidly filled up with healthy granulations, and in three weeks after the operation she was discharged cured. The ligature of the carotid came away on the 30th of December, and in the first week of January she returned to her friends in Bucks County, highly pleased with the results of the operation.

